# The Role of Intrinsic Pathway in Apoptosis Activation and Progression in Peyronie's Disease

**DOI:** 10.1155/2014/616149

**Published:** 2014-08-13

**Authors:** Carla Loreto, Giampiero La Rocca, Rita Anzalone, Rosario Caltabiano, Giuseppe Vespasiani, Sergio Castorina, David J. Ralph, Selim Cellek, Giuseppe Musumeci, Salvatore Giunta, Rados Djinovic, Dragoslav Basic, Salvatore Sansalone

**Affiliations:** ^1^Department of Bio-Medical Sciences, Anatomy and Histology Section, University of Catania, Via S. Sofia 87, 95123 Catania, Italy; ^2^Department of Experimental Biomedicine and Clinical Neuroscience, University of Palermo, 90100 Palermo, Italy; ^3^Department G.F. Ingrassia Section of Anatomic Pathology University of Catania, 95123 Catania, Italy; ^4^Department of Urology, School of Medicine Tor Vergata University of Rome, 00133 Rome, Italy; ^5^University College London Hospital, London WC1E 6BT, UK; ^6^Cranfield Health Cranfield University, MK43 0AL, UK; ^7^Sava Perovic Foundation, Center for Genito-Urinary Reconstructive Surgery, 11000 Belgrade, Serbia; ^8^Clinic of Urology, Clinical Center Nis, 18000 Nis, Serbia

## Abstract

Peyronie's disease (PD) is characterized with formation of fibrous plaques which result in penile deformity, pain, and erectile dysfunction. The aim of this study was to investigate the activation of the intrinsic apoptotic pathway in plaques from PD patients. Tunica albuginea from either PD or control patients was assessed for the expression of bax, bcl-2 and caspases 9 and 3 using immunohistochemistry and by measurement of apoptotic cells using TUNEL assay. Bax overexpression was observed in metaplastic bone tissue, in fibroblasts, and in myofibroblast of plaques from PD patients. Little or no bcl-2 immunostaining was detected in samples from either patients or controls. Caspase 3 immunostaining was very strong in fibrous tissue, in metaplasic bone osteocytes, and in primary ossification center osteoblasts. Moderate caspase 9 immunostaining was seen in fibrous cells plaques and in osteocytes and osteoblasts of primary ossification centers from PD patients. Control samples were negative for caspase 9 immunostaining. In PD patients the TUNEL immunoassay showed intense immunostaining of fibroblasts and myofibroblasts, the absence of apoptotic cells in metaplasic bone tissue and on the border between fibrous and metaplastic bone tissue. Apoptosis occurs in stabilized PD plaques and is partly induced by the intrinsic pathway.

## 1. Introduction

Peyronie's disease (PD) is a connective tissue disorder where formation of fibrous plaques in tunica albuginea (TA) and erectile tissue can result in penile deformity, pain, and erectile dysfunction [1–4]. Although PD is considered a localized disorder, a recent biomolecular study seems to suggest the involvement of the entire TA [5]. Fibrosis, its major pathological manifestation, arises from fibroblast proliferation and accumulation of extracellular matrix; PD progresses with formation of plaques or even ectopic calcification having the appearance of scar tissue, which prevents TA expansion during erections [6, 7]. An external stress, sustained most likely in the erect state (typically during sexual activity), has been suggested to initiate plaque development [8]; the resulting TA injury or tearing then is suggested to heal abnormally [9]. 

The mechanisms underpinning PD are unclear [9–11], and relatively little is known about the disease itself. As a result effective medical treatments that can alter its course or progression are not yet available [7, 12]. To date corrective surgery is the sole effective treatment. A greater understanding of PD pathophysiology at the molecular level has the potential to help develop novel medical therapeutic approaches.

**Figure 1:**
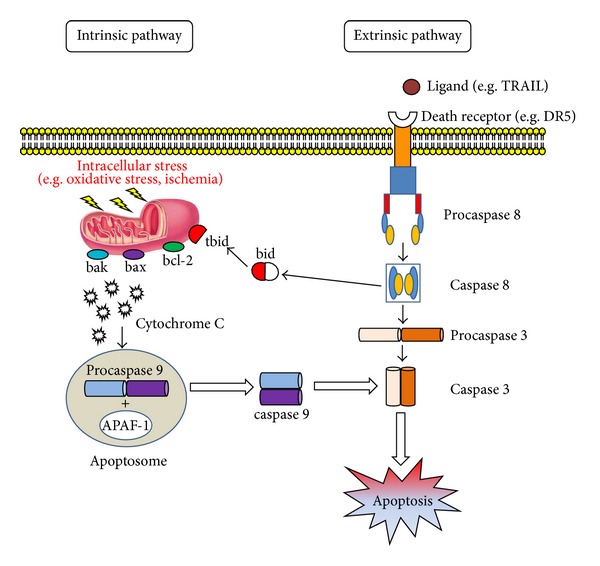
Apoptosis works through two main, alternative pathways: death receptor-mediated (or extrinsic) and mitochondria-dependent (or intrinsic). The former pathway is initiated by ligation of specific death receptors by their ligands. The main death receptors—Fas and tumour necrosis factor- (TNF-) related apoptosis inducing ligand (TRAIL) receptors DR4 and DR5—induce cell death following ligation with Fas ligand (FasL) or TRAIL, respectively, followed by recruitment of procaspase 8. This process gives rise to caspase 8 activation. The latter induces apoptosis by directly activating caspase 3 or by cleaving bid (BH3 interacting domain death agonist), resulting in mitochondrial dysfunction and subsequent release of cytochrome C and activation of caspases 9 and 3. Caspase 3 promotes the typical apoptosis features, including DNA fragmentation and cell death in several tissues. The mitochondrial pathway is partly influenced by bcl family members bound to the mitochondrial membrane, including bax and bcl-2, which are, respectively, pro- or antiapoptotic regulatory proteins. The antiapoptotic proteins bcl-2 and bcl-XL inhibit cytochrome c release, whereas bcl-2—associated X protein(bax), bcl-2 homologous antagonist/killer (bak), and bid, all proapoptotic proteins, promote its release from mitochondria. Cytochrome C and deoxyadenosine triphosphate (dATP) bind to apoptotic protease activating factor (APAF-1) to form a multimeric complex that recruits and activates procaspase 9, an apoptosis-mediating executioner protease that in turn activates caspase 3, resulting in cell apoptosis.

Apoptosis, or programmed cell death, is a fundamental mechanism with a key role throughout development [13]. In the fully formed organism it mediates the maintenance of tissue homeostasis involved in normal tissue turnover, whereas its dysregulation plays a role in multiple disease processes [14]. Apoptosis can take place either through the extrinsic (death receptor-mediated) or the intrinsic (mitochondrial) pathway (Figure 1). The former pathway involves activation of death signaling ligands like tumor necrosis factor-related apoptosis-inducing ligand (TRAIL) through its death receptors DR4 and DR5 and has a role in immune and homeostasis processes [13, 15]; these events in turn activate the initiator caspase 8, which cleaves and activates the executioner caspase 3. Executioner caspases induce biochemical and morphological changes such as chromatin condensation, nuclear fragmentation, and cytoskeletal degradation; once they have been activated the apoptosis process becomes irreversible.

The intrinsic mitochondrial pathway is activated by a range of exogenous and endogenous stimuli including DNA damage, ischemia, and oxidative stress (Figure 1). Moreover it plays an important function in development and in elimination of damaged cells [13]. The intrinsic pathway is influenced by members of the bcl family bound to the mitochondrial membrane, including bax and bcl-2, which act as pro- or antiapoptotic regulatory proteins, respectively [16]. In the intrinsic pathway the functional consequence of proapoptotic signaling is mitochondrial membrane perturbation and release of cytochrome C in the cytoplasm, where it forms a complex or apoptosome with apoptotic protease activating factor 1 (APAF1) and the inactive form of caspase 9. This complex hydrolyzes adenosine triphosphate to cleave and activate caspase 9. The initiator caspase 9 then cleaves and activates the executioner caspases 3, 6, and 7, resulting in cell apoptosis [14, 17]. The antiapoptotic proteins bcl-2 and bcl-XL inhibit cytochrome C release [18].

We have previously described apoptotic cell death in TA plaques from patients with PD and demonstrated its activation via the extrinsic pathway by assessing an overexpression of tumour necrosis factor-related apoptosis-inducing ligand (TRAIL) and its death receptor, DR5, in fibroblast and myofibroblast cells [19]. Following up on this previous work, we have decided to investigate the possible activation of the intrinsic pathway through the study of bax, bcl-2, caspase 9, the activation of caspase 3 (the point of no return leading irreversibly to cell death), and the TUNEL assay, which documents DNA fragmentation and hence the achievement of the apoptosis process.

## 2. Materials and Methods

### 2.1. Patients and Tissues

Wedge-shaped biopsy specimens (approximately 3 × 5 mm) were collected from 15 patients (mean age 53 ± 10 years; range 31–67) at the level of the corporotomy during corrective surgery for PD. All patients, who had had stable PD for at least 6 months, underwent albugineal grafting using the geometrical principle as originally described by Egydio and Sansalone [20]. The TA was incised and grafted at the level of the maximum penile curvature, where plaque formation was most prominent [21].

The study protocol was approved by the ethics committee of the Clinic of Urology, Clinical Center Nis, Nis, Serbia. The informed consent of each patient was obtained before tissue collection.

During preoperative examination all patients reported having spontaneous erections but being prevented from regular sexual intercourse by a penile curvature of above 45°. Degree of penile curvature and rigidity were evaluated with Doppler ultrasonography after intracavernous injection of 10–20 *μ*g alprostadil.

Control samples were specimens from 4 patients (mean age 23 ± 3 years; range 21–27) with congenital penile curvature who underwent Nesbit's corrective procedure [22]. Their clinical history was negative for generalized penile disease; none had macroscopic signs of degenerative or inflammatory disorders. Histological examination using Mayer's hematoxylin (Histolab Products AB, Sweden) and eosin (Histolab Products AB, Sweden) showed no detectable pathological abnormalities (not shown). 

### 2.2. Immunohistochemistry

For the immunohistochemical studies, PD plaques were fixed overnight in 10% neutral buffered formalin (Bio-Optica, Italy). After fixation and overnight washing in water they were dehydrated in graded ethanol and paraffin-embedded. They were then cut into 5 *μ*m thick sections, using a microtome (Hn40, Reichert-Jung, USA) and placed on silanized glass slides. After rehydration endogenous peroxidase activity was quenched by treatment with 3% H_2_O_2_ for 10 min as previously described [23]. Nonspecific antibody binding was blocked by treatment with normal horse/goat serum diluted 1 : 20 in phosphate buffered saline (PBS), 0.1% bovine serum albumin (BSA, Roche Applied Science, Germany). Sections were treated (5 min × 3 times) in capped polypropylene slide-holders with citrate buffer (pH 6) using a microwave oven (750 W) to unmask antigen sites.

The following primary antibodies were used: rabbit polyclonal antihuman bax (1 : 100, Dako, Denmark), mouse monoclonal antibody antihuman bcl-2 (Dako, Denmark), mouse monoclonal antihuman caspase 9 (1 : 100, Santa Cruz Biotechnology, USA), and rabbit monoclonal antihuman caspase 3 (1 : 50, Abcam, UK). The primary antibodies were applied directly onto sections and slides were incubated overnight (4°C) in a humid chamber. The sections were then washed in PBS and treated with a biotinylated antibody and detected using peroxidase-labeled streptavidin, both incubated for 10 min at room temperature (LSAB+System-HRP, Dako Italy).

The immunoreaction was assessed using a Zeiss Axioplan light microscope (Oberkochen, Germany) after incubating sections in 0.1% 3,3′-diaminobenzidine and 0.02% hydrogen peroxide solution (DAB substrate kit, Vector Laboratories, Burlingame, CA, USA) for 4 min. Sections were lightly counterstained with Mayer's hematoxylin and then mounted on GVA mount (Zymed Laboratories, San Francisco, CA, USA).

### 2.3. Evaluation of Immunohistochemical Staining

Staining for bax, bcl-2, caspase 9, and caspase 3 was classified as negative/positive. Immunohistochemical staining was brown chromogen detected on the edge of the hematoxylin-stained cell nucleus or distributed in the cytoplasm or the membrane. Intensity of staining (IS) was graded in a semiquantitative manner using a 5-point scale: 0 = no detectable staining, 1 = weak staining, 2 = moderate staining, 3 = strong staining, and 4 = very strong staining. The proportion of immunopositive cells (extent score = ES) was evaluated at 20x magnification blindly by two anatomists and a histologist. ES was scored as a proportion of the final number of 100 cells into five classes: <5% (0), 5–30% (+), 31–50% (++), 51–75% (+++), and >75% (++++). Counting was performed at 20x magnification.

Positive and negative controls were run to test the specific reaction of the primary antibodies used. Positive controls were basal cell carcinoma specimens. For the negative controls, randomly selected sections from PD patients were treated with normal rabbit serum instead of the specific antibodies.

### 2.4. In Situ Detection and Measurement of Apoptotic Cells (TUNEL)

In situ detection of apoptosis at the single cell level was performed by terminal deoxynucleotidyl transferase- (TdT-) mediated dUTP-biotin nick end labeling, TUNEL (In Situ Cell Death Detection Kit, POD, Roche) as previously described [24]. The method involves adding deoxyuridine triphosphate (dUTP) labeled with fluorescein to the ends of the DNA fragments by the catalytic action of TdT. End-labeling experiments were performed in triplicate to enable standardization of the results for the different tissue samples. Paraffin-embedded sections of 5 *μ*m thicknesses were dewaxed. Slides were rinsed twice in 0.01 M PBS (pH 7.4), transferred to 0.07 M citrate buffer (pH 6.0; Bio-Optica), and treated in a microwave oven at 750 W for 1 min for permeabilization. Sections were then immersed in Tris-HCl 0.1 M (Roche), pH 7.5, containing 3% BSA and 20% normal bovine serum (Sigma-Aldrich, St. Louis, MO, USA) for 30 min at 20°C, rinsed twice in PBS, and immersed in TdT buffer (Roche). Sections were then covered with TdT and fluorescein-labeled dUTP in TdT buffer and incubated in a dark humid chamber at 37°C for 60 min. They were incubated with an antibody specific for fluorescein conjugated to peroxidase with 30 min incubation at 37°C. Staining was visualized with DAB, which stains nuclei with DNA fragmentation brown. Sections were counterstained with Mayer's hematoxylin. In negative controls TdT was omitted from the reaction. Ten fields from randomly selected slides were observed under a light microscope. Each field was photographed with a digital camera (Canon, Japan) at 20x and 40x magnification. On each photomicrograph the same three observers, blinded to sample identity, counted the cells exhibiting a positive TUNEL reaction. The proportion of positive cells was calculated for each photomicrograph and a mean value was obtained for each sample.

### 2.5. Computerized Image Analysis

To quantify immunohistochemical staining, 10 sections/sample were analyzed in stepwise fashion as a series of consecutive fields with a 40x magnification; the stained area was expressed as pixels/field. Randomly selected fields from each section were analyzed and the percent area staining for bax, bcl-2, caspase 9, and caspase 3 was calculated using AxioVision rel. 4.8.2 image analysis software and an AxioVision 4 Module AutoMeasure (Zeiss, Göttingen, Germany) to quantify the level of immunolabeling in each field. Values from all consecutive images of each biopsy were averaged. Digital pictures were taken using an Axiocam camera (Zeiss, Göttingen).

### 2.6. Statistical Analysis

Statistical analysis was performed using SPSS software (rel. 16.0, Chicago, IL, USA). Comparisons between mean values were tested with Student's unpaired *t*-test. *P* values less than 0.05 were considered significant. Cohen's kappa coefficient was used to measure interobserver agreement and averaged over the three observers. Overall agreement was graded as follows: 0–0.2 (slight), 0.21–0.40 (fair), 0.41–0.60 (moderate), 0.61–0.80 (substantial), and 0.81–1.0 (almost perfect).

## 3. Results

### 3.1. General Observations

On hematoxylin and eosin-stained specimens the collagen fiber arrangement was mostly disrupted. Fibers were affected to different degrees in all PD patients, the damage involving fragmentation, tears, and splitting. Elastic fibers were often fragmented; clumps of collagen bundles and surrounding elastic fibers were also noted. In contrast, control TA samples exhibited preserved collagen bundles displaying longitudinal fiber orientation in the outer layer and circular fibers in the inner layer (not shown).

### 3.2. Bax Expression

**Figure 2:**
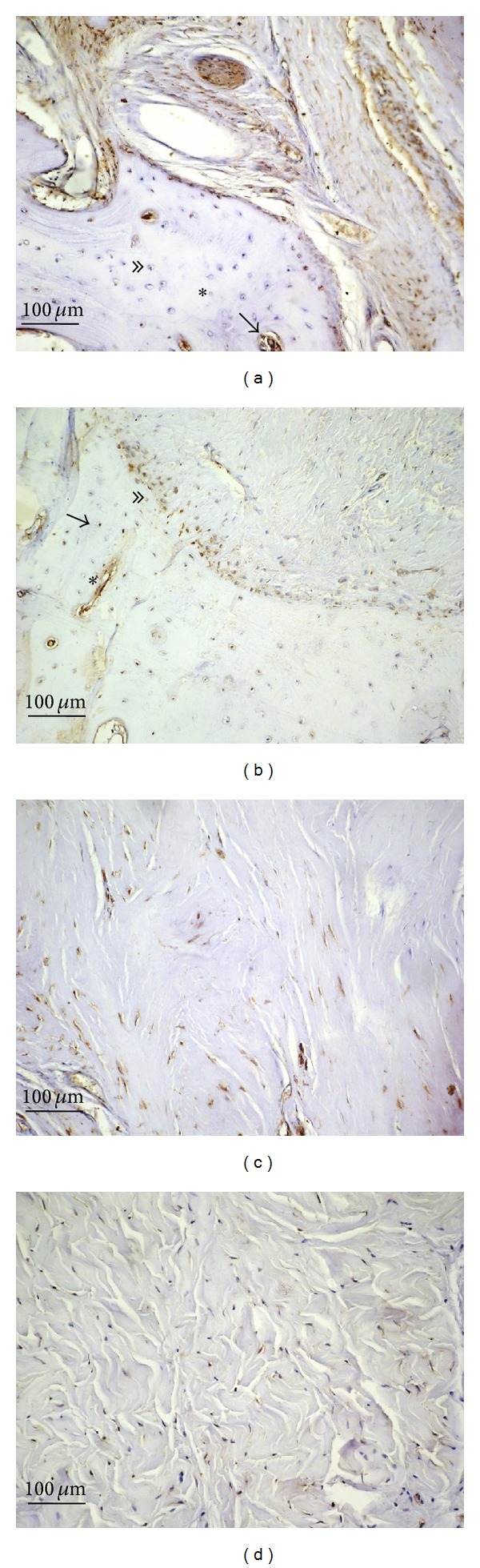
Bax immunostaining in plaque from a patient with Peyronie's disease (a, b, c) and in control tissue (d); magnification is 20x for all. Scale bar = 100 *μ*m. Asterisk at (a): areas of osseous metaplasia with heterotopic ossification; double arrowhead: an osteocyte in bone lacunae; arrow: bax overexpression in an osteoblast of a primary ossification center. Arrow at (b): bax overexpression in an osteocyte in metaplastic bone; double arrowhead: bax overexpression in osteoblasts and fibroblasts on the border of the metaplastic bone tissue; asterisk: bax overexpression in a blood vessel.

PD plaques showed areas of osseous metaplasia with heterotopic ossification (Figure 2(a), asterisk) and several osteocytes were detected in bone lacunae (Figure 2(a), double arrowhead).

Immunohistochemical examination of sections from PD plaques showed bax overexpression (IS: 4; ES: ++++) in some osteocytes in metaplastic bone (Figure 2(b), arrow) and in osteoblasts found in the primary ossification centers (Figure 2(a), arrow). In particular bax overexpression was detected in osteoblasts and fibroblasts on the border of the metaplastic bone tissue (Figure 2(b), double arrowhead) and in vessels (Figure 2(b), asterisk). Very strong bax positivity was also demonstrated in PD plaques in the fibroblast cytoplasm and in the myofibroblast membrane and cytoplasm (Figure 2(c)) (IS: 4; ES: ++++), whereas very few immunostained cells were detected in control samples (Figure 2(d)) (IS: 3; ES: +). The percentage of bax immunopositive cells in PD plaque and control tissue is shown in Figure 7(a). Interobserver agreement was 0.94.

### 3.3. Bcl-2 Expression

**Figure 3:**
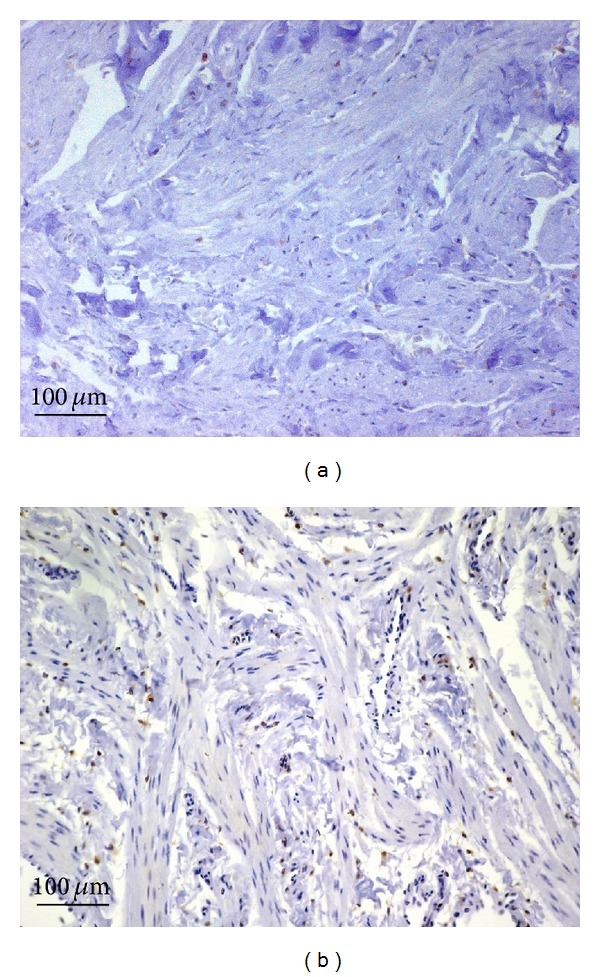
Bcl-2 immunostaining in plaque from a patient with Peyronie's disease (a) and in control tissue (b); magnification is 20x for all. Scale bar = 100 *μ*m.

Little or no bcl-2 immunostaining was detected in samples from PD patients (IS: 1; ES: 0) or from controls (IS: 2; ES: +) (Figures 3(a) and 3(b), resp.). The percentage of bcl-2 immunopositive cells in PD plaque and control tissue is shown in Figure 7(b). Interobserver agreement was 0.95.

### 3.4. Caspase 3 Expression

**Figure 4:**
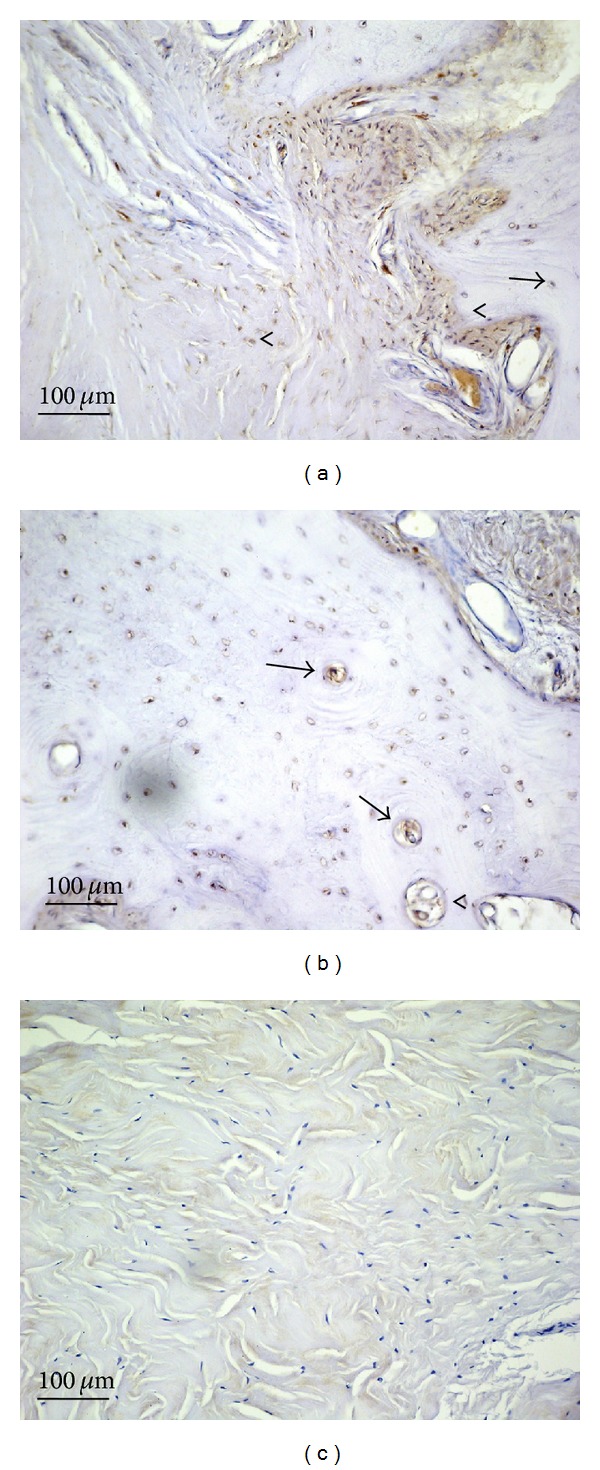
Caspase 3 immunostaining in plaque from a patient with Peyronie's disease ((a) and (b)) and in control tissue (c); magnification is 20x for all. Scale bar = 100 *μ*m. (a) Arrowheads: caspase 3 immunostaining in fibrous tissue; arrow: caspase 3 immunostaining in a metaplastic bone osteocyte. (b) Arrows: haversian canals; arrowhead: a primary ossification center.

Caspase 3 immunostaining was very strong (IS: 4; ES: ++++) in fibroblasts lying on the border between fibrous and metaplastic bone tissue; it was also very strong in fibrous tissue (Figure 4(a), arrowheads) and in metaplastic bone osteocytes (Figure 4(a), arrow). Haversian canals (Figure 4(b), arrows) exhibiting primary ossification centers (Figure 4(b), arrowhead) were observed in metaplastic bone wherein a strong caspase 3 immunostaining was found in osteoblasts. Little or no caspase 3 immunostaining was seen in control samples (IS: 3; ES: 0) (Figure 3(c)). The percentage of caspase 3 immunopositive cells in PD plaque and control tissue is shown in Figure 7(c). Interobserver agreement was 0.90.

### 3.5. Caspase 9 Expression

**Figure 5:**
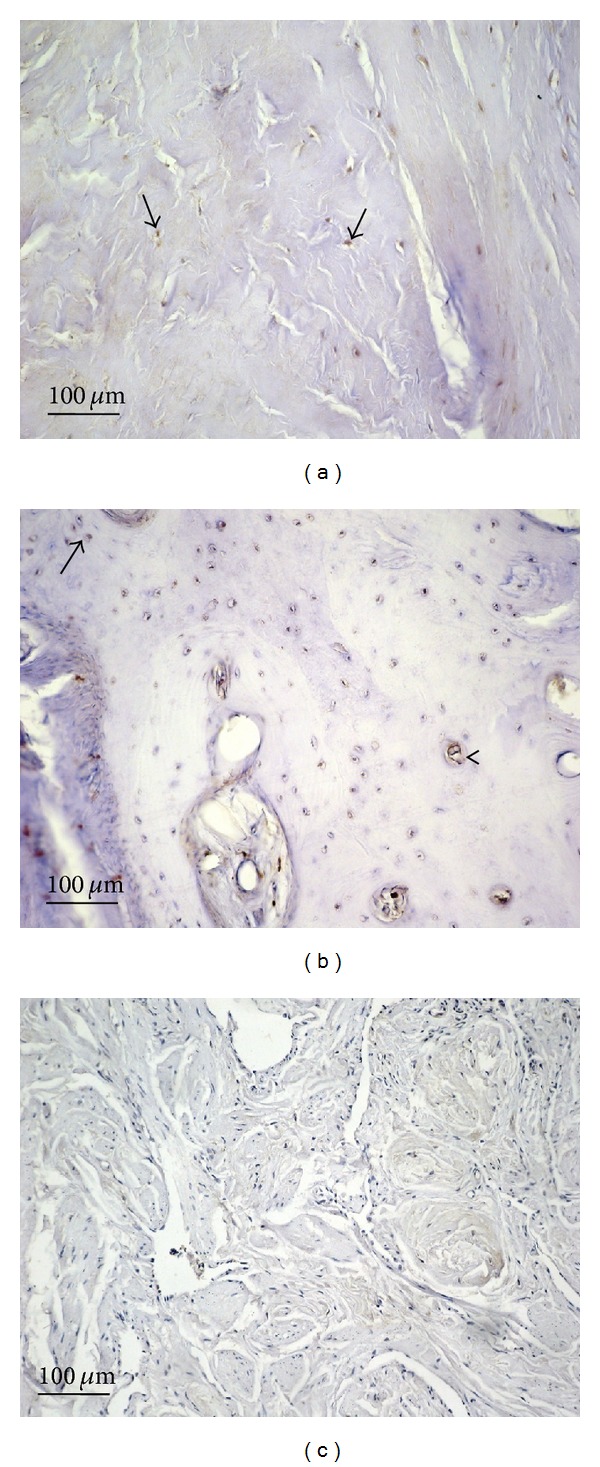
Caspase 9 immunostaining in plaque from a patient with Peyronie's disease (a and b) and in control tissue (c); magnification is 20x for all. Scale bar = 100 *μ*m. (a) Arrows: caspase 9 immunostaining in fibrous cells from PD plaques (Figure 5(a), arrows) and in osteocytes (arrow). (b) Arrowheads: caspase 9 immunostaining in osteoblasts of a primary ossification centers.

Moderate immunostaining for caspase 9 was seen in fibrous cells from PD plaques (Figure 5(a), arrows) and in osteocytes (arrow) and osteoblasts of primary ossification centers (arrowhead) (Figure 5(b)) (IS: 2; ES: +++). Control samples were negative for caspase 9 immunostaining (Figure 5(c)) (IS: 0; ES: 0). The percentage of bax immunopositive cells in PD plaque and control tissue is shown in Figure 7(d). Interobserver agreement was 0.89.

### 3.6. TUNEL Staining

**Figure 6:**
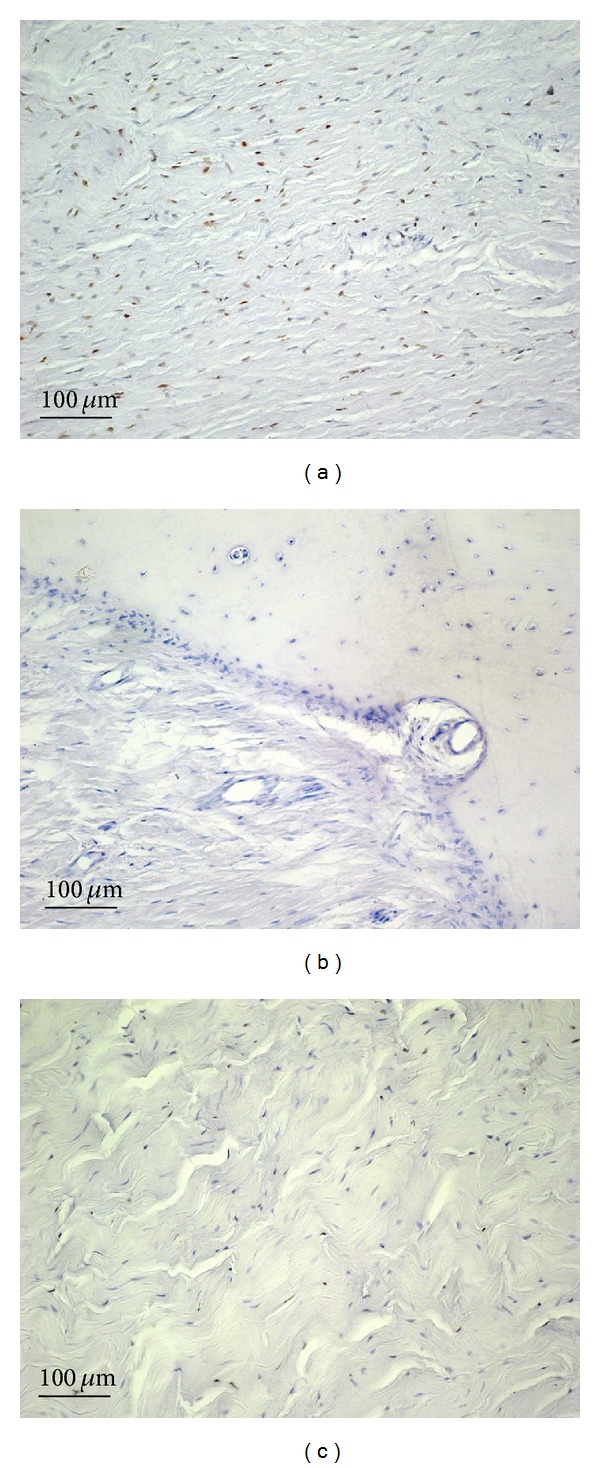
TUNEL staining in plaque from a patient with Peyronie's disease ((a) and (b)) and in control tissue (c); magnification is 20x for all. Scale bar = 100 *μ*m.

**Figure 7:**
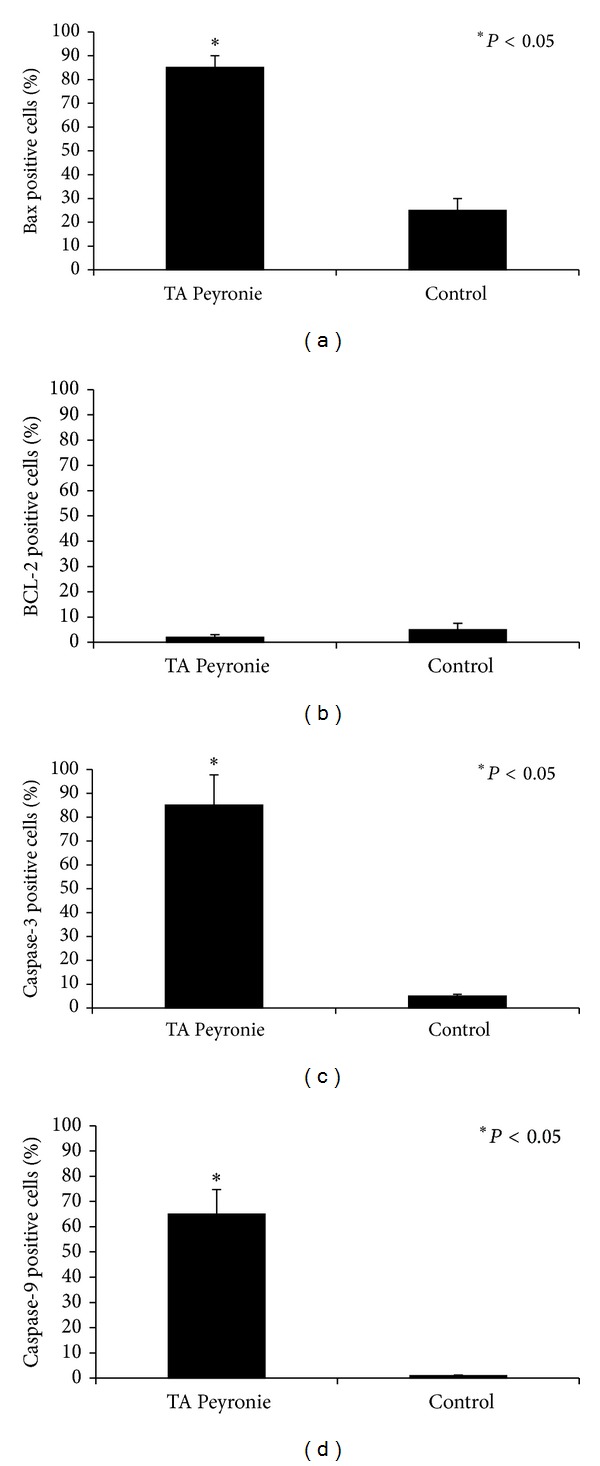
Quantitative analysis of bax (a), bcl-2 (b), caspase 3 (c), and caspase 9 (d) immunostaining in PD samples. In both panels, asterisks indicate significant differences versus the control group (*P* < 0.05).

**Figure 8:**
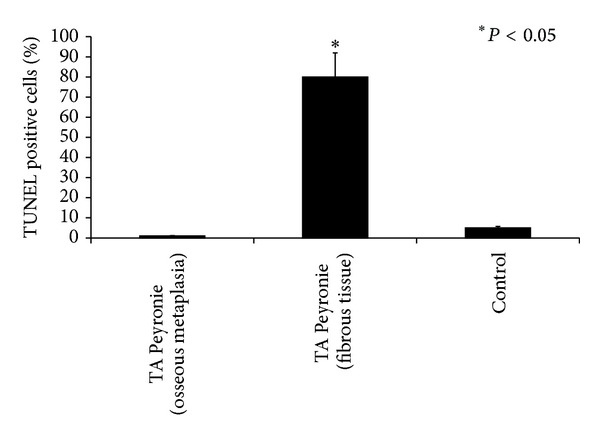
Quantitative analysis of TUNEL-positive cell staining in PD samples. Different cell positivity can be seen in areas with osseous metaplasia and in fibrous tissue areas. Asterisks indicate significant differences versus the control group (*P* < 0.01).

The TUNEL immunoassay showed intense staining of fibroblasts and myofibroblasts from PD plaques (Figure 6(a)) and no stained cells in the metaplastic bone tissue or on the border between fibrous and metaplastic bone tissue (Figure 6(b)). Very few apoptotic cells were detected in control specimens (Figure 6(c)). Quantitative analysis of TUNEL-positive cell staining in PD samples is reported in Figure 8.

## 4. Discussion

Even though PD has been described in the 18th century, its pathogenic mechanism and molecular basis are poorly understood and its therapeutic approach is still empirical [25].

A previous study by our group documented the involvement of the extrinsic pathway in the activation of programmed cell death in PD. The present study examines the possible activation of the intrinsic apoptotic pathway through the immunohistochemical demonstration of staining for a number of key molecules: bcl-2, bax, anti- and proapoptotic oncoproteins, respectively, involved in apoptosis regulation, modulating cell survival or death [26–30], caspase 9, which is associated with the intrinsic pathway, and the executioner caspase 3 (Figure 1). The study also seeks to demonstrate the completion of the apoptotic process using the TUNEL assay. Bax upregulation and negative bcl-2 immunostaining, associated with moderate caspase 9 activation, suggest that the apoptotic process documented in PD patients is at least partly regulated by the intrinsic pathway.

TA tissue from PD patients also exhibited upregulation of caspase 3, the executioner caspase of the apoptotic cascade. The apoptotic cascade has been subdivided into three sets of stages.* Initiation stages *include induction of the cascade, for instance, by ligand-receptor interactions or a cellular stress leading to the first proteolytic event.* Execution stages *begin with the activation of executioner caspases such as caspase 3: this is called the “point of no return” since, once activated, these proteases degrade a variety of proteins (including those involved in DNA maintenance and repair such as PARP), resulting in irreversible cell damage. These complex events lead to* apoptotic death*, with collapse of the nucleus and of the cell itself and DNA fragmentation [31].

The caspase 3 immunopositivity found in the plaques from the PD patients, both in fibrous and in metaplastic bone tissue, seems to be in contrast with the TUNEL test results, which suggest that apoptotic cells are found in fibrous tissue but are absent at the level of the osseous metaplasia. However, the discrepancy can be accounted for by the fact that the anticaspase 3 antibody used in these experiments recognizes both the cleaved and the noncleaved form of the protein. This suggests that whereas the apoptotic process takes place in fibrous tissue as a defense mechanism, at the level of the osseous metaplasia, where a degenerative process is already under way, the apoptosis mechanism is already activated but stops and does not continue with caspase 3 activation (cleavage). Apoptosis can therefore be regarded as an injury-limiting mode of cell disposal [32]. Indeed osseous metaplasia can be considered as a tissue adaptation event responding to a microenvironmental change, leading to replacement of sensitive cells with less sensitive ones. Areas of fibrous tissue subject to chronic trauma and a hypoxic microenvironment may therefore give rise to formation of bone tissue presumably from stem cells that undergo osteogenic differentiation [33]. TA disruption in PD usually involves a variety of steps that include a local increase in microvascular permeability, persistent accumulation of fibrin and collagen, perivascular inflammation, elastic fiber disruption and loss, disruption of collagen bundle organization, increased synthesis of transforming growth factor *β*1 (TGF-*β*1), and ultimately calcification followed by ossification [34–39]. Increased expression of TGF-*β*1, plasminogen activator inhibitor 1, reactive oxygen species (ROS), and other profibrotic factors as well as of inducible nitric oxide synthase (iNOS) leads to fibroblast/myofibroblast proliferation and collagen buildup [40, 41] as a defense mechanism against oxidative stress. ROS, iNOS, and TGF-*β*1 are capable of inducing fibroblast and myofibroblast apoptosis [33, 42–46]. In normal repair and healing a protective mechanism clears myofibroblasts through apoptosis; however, failure of the mechanism entails their persistence inducing fibrosis, collagen buildup, and tissue contraction [7]. Activation of the apoptosis mechanism through the extrinsic pathway, demonstrated in our previous paper, and via the mitochondrial pathway in the present one may be regarded as the reason for plaque stabilization and the halting of fibrosis progression. However, after activation the apoptosis process could be blocked by endogenous apoptosis inhibitors, as likely occurs in the osseous metaplasia area. Further in vivo and in vitro research are therefore needed to gain insights into this delicate process.

A study by Lucattelli et al. [47] demonstrated in a new mouse model of PD that mice aged 12 months develop osseous metaplasia, probably as a consequence of upregulation of hypoxia-inducible factor-1 (HIF-1): this seems to determine an increased expression of HIF-1 target genes such as TGF-*β* and iNOS which, as noted above, are partly responsible for apoptosis induction. This is probably why there were no apoptotic cells in the osseous metaplasia region.

A further consideration emerging from the analysis of our data is that the moderate expression of caspase 9 and overexpression of caspase 3 indicate a weak role for the intrinsic pathway in programmed cell death, a role that is played principally by the extrinsic pathway as demonstrated by our previous findings. This is reasonable, since this pathway is basically activated by death receptor binding with ligands such as TRAIL, whereas the intrinsic pathway is activated by exogenous stimuli such as inflammation-induced oxidative stress (ROS) [40].

Recent studies have shown that phosphoinositide 3-kinase (PI3K)/Akt signaling regulates fibrotic responses including collagen synthesis and cell proliferation [48]. Jung and colleagues synthesized HS-173, a novel PI3K inhibitor, and found that it inhibited fibroblast growth in a dose-dependent manner and induced apoptosis [48]. Although these findings appear to contrast with ours, the discrepancy may be ascribed to the fact that they were obtained in vivo or that our samples contained stabilized plaque, likely through apoptosis activation and achievement. Indeed it is well known that when cells lack proliferation factors due, for instance, to tissue overgrowth, they stop proliferating and often die by apoptosis [14].

Previous studies have advanced the hypothesis of an abnormal wound healing process in PD, possibly through a failure of fibroblast apoptosis [49]. Alternatively the significant increase in the number of apoptotic cells detected in PD plaques could be ascribed to an efficient mechanism of programmed cell death leading to plaque stabilization; it may therefore be hypothesized that although the apoptosis process could be impaired during disease evolution, it might however result in plaque stabilization. It would therefore be interesting to study nonstabilized plaque to gain further insights into the activation and progression of the apoptosis process.

In this study we tested the involvement of the intrinsic apoptosis pathway and of executioner caspases in PD and completed the experimental work with the TUNEL assay. Novel therapeutic strategies, for instance, based on TRAIL agonists, could help PD patients through enhancement of myofibroblast death in the attempt to induce plaque stabilization and prevent disease progression.

## 5. Conclusion

Apoptotic cell death occurs in stabilized PD plaques and is partly induced by the intrinsic mitochondrial pathway. The present findings can have clinical implications and may help devise improved treatment strategies. A therapeutic approach aimed at enhancing apoptosis-inducing molecules would at least help delay the progression of PD. Identification of target molecules for gene construct or biological or chemical reagent delivery to target sites could contribute to inducing PD plaque stabilization.
